# Analyzing the Impact of Vaccine Availability on Alternative Supplier Selection Amid the COVID-19 Pandemic: A cFGM-FTOPSIS-FWI Approach

**DOI:** 10.3390/healthcare9010071

**Published:** 2021-01-13

**Authors:** Toly Chen, Yu-Cheng Wang, Hsin-Chieh Wu

**Affiliations:** 1 Department of Industrial Engineering and Management, National Chiao Tung University, University Road, Hsinchu 1001, Taiwan; tolychen@ms37.hinet.net; 2 Department of Aeronautical Engineering, Chaoyang University of Technology, Taichung 41349, Taiwan; 3 Department of Industrial Engineering and Management, Chaoyang University of Technology, Taichung 41349, Taiwan; hcwul@cyut.edu.tw

**Keywords:** COVID-19 pandemic, alternative supplier, fuzzy collaborative intelligence, wafer fabrication

## Abstract

The supply chain disruption caused by the coronavirus disease 2019 (COVID-19) pandemic has forced many manufacturers to look for alternative suppliers. How to choose a suitable alternative supplier in the COVID-19 pandemic has become an important task. To fulfill this task, this research proposes a calibrated fuzzy geometric mean (cFGM)-fuzzy technique for order preference by similarity to ideal solution (FTOPSIS)-fuzzy weighted intersection (FWI) approach. In the proposed methodology, first, the cFGM method is proposed to accurately derive the priorities of criteria. Subsequently, each expert applies the FTOPSIS method to compare the overall performances of alternative suppliers in the COVID-19 pandemic. The sensitivity of an expert to any change in the overall performance of the alternative supplier is also considered. Finally, the FWI operator is used to aggregate the comparison results by all experts, for which an expert’s authority level is set to a value proportional to the consistency of his/her pairwise comparison results. The cFGM-FTOPSIS-FWI approach has been applied to select suitable alternative suppliers for a Taiwanese foundry in the COVID-19 pandemic.

## 1. Introduction

The coronavirus disease 2019 (COVID-19) pandemic has forced many factories to close [1,2,3], causing related supply chains to break [3,4]. For example, the main production area of the spray nozzles of disinfection sprayers is in China, one of the regions where the cases of the COVID-19 pandemic were first identified. As a result, manufacturers of disinfection sprayers could not assemble and ship them in a short time. Automakers were also forced to postpone the delivery of vehicles due to the temporary closure of parts suppliers to slow the spread of COVID-19 [5]. In order to make up for the shortage of raw materials, manufacturers need to find alternative suppliers [6]. Therefore, how to select suitable alternative suppliers for manufacturers amid the COVID-19 pandemic has become a key task [7]. This task can be regarded as a fuzzy group multi-criteria decision-making problem [8,9] for the following reasons. First, it is clear that there are many uncertainties associated with the COVID-19 pandemic, which are mainly caused by the human intervention [10]. Fuzzy sets, such as ordinary fuzzy sets [11,12], intuitionistic fuzzy sets [13], interval type-2 fuzzy sets [14], and hesitant fuzzy sets [15], are particularly useful for dealing with this type of uncertainty. Second, the impact of the COVID-19 pandemic is so serious that it requires multiple experts to make a joint decision to avoid personal bias and omissions [16]. Furthermore, when making such a decision, there are many criteria that need to be considered [17]. Some are operational, some are health-related, and others are specific to the COVID-19 pandemic. Panetta [18] classified criteria that need to be considered in making decisions amid the COVID-19 pandemic into three categories: traditional business value related criteria, crisis and disruption related criteria, and social and emotional criteria.

Although a manufacturer usually has multiple suppliers, most of these suppliers are likely to be affected by the COVID-19 pandemic. In addition, the surviving suppliers may not be able to provide sufficient supplies for the manufacturer. Therefore, the manufacturer needs to find alternative suppliers. In order to select suitable alternative suppliers for a manufacturer amid the COVID-19 pandemic, a fuzzy collaborative intelligence approach is proposed in this study. Issues related to health care has become a key consideration when making such selections amid the COVID-19 pandemic. For example, alternative suppliers should be located in regions with better health care to protect the health of workers in our factory. The proposed methodology consists of three main parts. The first part is the calibrated fuzzy geometric mean (cFGM) method, which is a modification of the prevalent fuzzy geometric mean (FGM) method [19,20,21], for deriving the priorities of criteria, which are essential for selecting a suitable alternative supplier. The traditional FGM method is not accurate enough. After tuning the membership function of each fuzzy priority, the cFGM method can improve the accuracy. Subsequently, the fuzzy technique for order preference by similarity to ideal solution (FTOPSIS) method [8,9,14,22] is applied by each expert to compare the overall performances of alternative suppliers amid the COVID-19 pandemic. The sensitivity of each expert to changes in the overall performance of an alternative supplier is also considered. However, the evaluation results by different experts are not equal and need to be aggregated. To this end, the fuzzy weighted intersection (FWI) operator [21,22] is applied to aggregate the evaluation results by all experts. Compared with existing aggregators (such as fuzzy extent analysis (FEA) [23], FGM, and fuzzy intersection (FI) [24,25]), FWI considers the inequality of experts’ authorities in a better way, and generates results that are acceptable to all experts [26]. Finally, since the presence or absence of a COVID-19 vaccine will affect experts’ decision-making results, this study considers two possible scenarios: vaccines already available and no vaccines yet.

The differences between the proposed methodology and some existing methods are summarized in Table 1.

The remainder of this paper is organized as follows. Section 2 is dedicated to the literature review. Section 3 introduces the proposed fuzzy collaborative intelligence approach for selecting a suitable alternative supplier amid the COVID-19 pandemic. Section 4 reports the results of applying the fuzzy collaborative intelligence approach to select suitable alternative suppliers for a wafer foundry in Taiwan amid the COVID-19 pandemic. Some existing methods are also applied to this case for comparison. Section 5 concludes this study and puts forth some future research topics.

## 2. Literature Review

### 2.1. Impact of the COVID-19 Pandemic on Suppliers

The COVID-19 pandemic forced some suppliers to close. As a result, the contracts signed with these suppliers were canceled, breached, or ignored. Purchasers have no choice but to find an alternative supplier. Supplier relationships have encountered unprecedented challenges [30]. In the view of Howorth [31], supplier relationships will change from short-term trading nature to long-term collaborative and strategic relationships. In addition, the competition for the limited capacity of alternative suppliers in the upstream or midstream segments of supply chains will intensify [6]. To explore this topic, Ivanov [32] conducted a simulation study to evaluate and predict the impact of the COVID-19 pandemic on a global supply chain. Hoek [33] analyzed seven companies that adopted total costs of ownership, supplier segmentation, and supply chain change management theory to mitigate the impact of the COVID-19 pandemic. According to the experimental results, finding alternative suppliers is becoming more and more important for reducing risks in the supply chain. Sharma et al. [34] developed a framework for enhancing the survivability of supply chains within and after the COVID-19 pandemic by optimizing six criteria: performance under uncertainty, configuration, governance works, viability, collaboration, and digital data-driven. Majumdar et al. [35] used a case to highlight the fragility of clothing supply chains in South Asian countries amid the COVID-19 pandemic. Then, they suggested that the disruption risk sharing agreement should be included in the procurement contract, and suppliers should be prohibited from subcontracting without authorization. Ivanov and Dolgui [36] discussed the integrity and viability of an intertwined supply network composed of companies that play different roles (buyer or supplier) in different supply chains. It is believed that amid the COVID-19 pandemic, intertwined supply networks can provide more sustainable services to society than other types of supply chains.

### 2.2. Alternative Supplier Selection amid the COVID-19 Pandemic

Supplier selection is a multi-criteria decision-making problem, which has been extensively studied [37,38,39,40]. Many factors are considered to be essential for choosing the right supplier, such as product quality, price, delivery speed, company reputation, supplier relationship, etc. [37,38,39,40]. In contrast, past research rarely discussed health-related factors directly, but regarded them as part of sustainability. For example, in the view of Mani et al. [41], suppliers with social sustainability should perform well in equity, health, safety, wages, education, philanthropy, and child and bonded labor. In addition, safety and health are one of the five dimensions of supplier social sustainability, which are crucial to the performance of a supply chain [20]. However, a supplier’s performance in these aspects may not be known.

**Figure 1:**
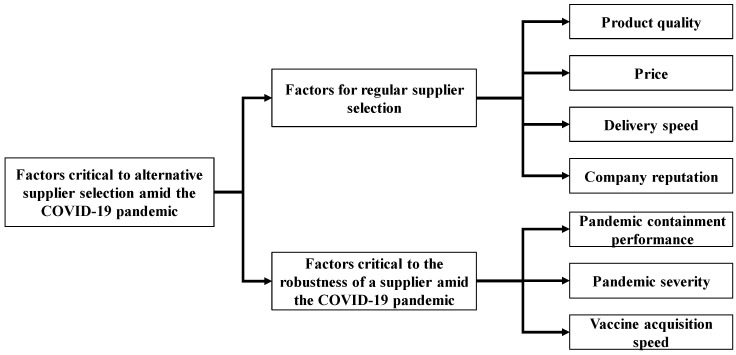
Factors critical to the selection of an alternative supplier amid the coronavirus disease 2019 (COVID-19) pandemic.

The COVID-19 pandemic has caused many supply chains to break [3,4]. Manufacturers located in the midstream or downstream of such supply chains were forced to find alternative suppliers [6]. Alternative suppliers are suppliers with which a formal partnership does not exist. In regular supplier selection, alternative suppliers may not be the best choice, but must be resorted to during the COVID-19 pandemic. Therefore, the criteria for selecting alternative suppliers may be different from those for selecting regular suppliers [7,42]. In addition, amid the COVID-19 pandemic, manufacturers (including suppliers and alternative suppliers) must remain robust to the pandemic to ensure their operations [17,41]. Factors that are critical to the robustness of a manufacturer to the COVID-19 pandemic include pandemic containment performance, pandemic severity, vaccine acquisition speed, demand shrinkage, supplier impact, and infection risk [43]. Some of these factors should also be taken into account when choosing an alternative supplier amid the COVID-19 pandemic. In short, at least seven factors are considered critical to the selection of an alternative supplier amid the COVID-19 pandemic: product quality, price, delivery speed, company reputation, pandemic containment performance, pandemic severity, and vaccine acquisition speed (see Figure 1). Supplier impact and demand shrinkage have a greater impact on downstream assemblers. If there is a vaccine, the risk of infection can be controlled. For these reasons, the three factors (supplier impact, demand shrinkage, and vaccine acquisition speed) are not included, and two scenarios (with and without vaccines) are analyzed. In addition, during the COVID-19 pandemic, most regional governments control the entry and exit of people. In order to avoid an impact on manufacturers, the import and export of raw materials are relatively unlimited. Therefore, the selection of alternative suppliers is less affected by relevant government regulations.

### 2.3. Decision-Making amid the COVID-19 Pandemic

There are many uncertainties and risks in making decisions amid the COVID-19 pandemic [44,45]. To tackle this, some fuzzy or probabilistic decision-making methods [46,47,48] have been proposed. For example, Wu et al. [10] proposed a fuzzy collaborative intelligence-based fuzzy analytic hierarchy process (FAHP) approach to evaluate and compare fifteen intervention strategies in response to the COVID-19 pandemic. In their methodology, each expert applies the FGM method to evaluate the relative priorities of criteria. Then, the layered partial consensus approach [49] is applied to aggregate the evaluation results of most experts. Finally, the generalized fuzzy weighted assessment approach is proposed to evaluate the effectiveness of an intervention strategy for tackling the COVID-19 pandemic. Chen and Lin [17] proposed a FAHP method for comparing various smart and automation technology applications to ensure the long-term operation of a factory amid the COVID-19 pandemic. Chen et al. [43] proposed a fuzzy collaborative intelligence method to evaluate the robustness of a factory to the COVID-19 pandemic. Fuzzy intersection and partial-consensus fuzzy intersection were applied to aggregate experts’ evaluation results, depending on the number of experts who have reached a consensus. Fong et al. [44] conducted Monte Carlo simulations to extrapolate several time series data of the COVID-19 pandemic. Then, they built a deep learning network to predict these time series. Based on these predictions, a fuzzy inference system (FIS) was developed to analyze the trend of the COVID-19 pandemic. Wu et al. [50] applied machine learning technologies [51,52] to assess the severity risk of an incoming patient, thereby making decisions in the allocation of medical resources. Govindan et al. [53] developed a FIS for assisting demand management in a healthcare supply chain. To investigate the impact of the COVID-19 pandemic on family investment decisions, Yue et al. [54] applied linear probability and probit models. The experimental results showed that households who knew someone infected with COVID-19 lost confidence in the economy and might become risk-averse. Burlea-Schiopoiu and Ferhati [55] applied the structural equation modeling (SEM) method to identify the factors critical to the performance of a healthcare sector, thereby defining key performance indexes. The COVID-19 pandemic has impacted many industries. Yu et al. [56] proposed a similar SEM method to identify factors that may influence people’s fear of missing out, thereby guiding people’s decisions to repost news related to the COVID-19 pandemic on social media. Lystad et al. [57] fitted seasonal autoregressive integrated moving average (ARIMA) models to predict the utilization of manual therapy services in Australia. The decline is expected upon utilization, providing valuable information for service providers and governments to consider in making responsive decisions.

## 3. The Fuzzy Collaborative Intelligence Approach

The fuzzy collaborative intelligence approach used to select suitable alternative suppliers amid the COVID-19 pandemic comprises three main parts: the cFGM method for determining the priorities of criteria, the FTOPSIS method for comparing the overall performances of alternative suppliers, and the FWI operator for aggregating the comparison results by all experts. The three parts are described in the following subsections. 

The steps for implementing the fuzzy collaborative intelligence approach are as follows:

Step 1. (Each expert) Determine the priorities of criteria using cFGM.
Step 2. (Each expert) If the critical ratio is less than 0.1, go to Step 3; otherwise, modify the comparison matrix and return to Step 1.
Step 3. (Each expert) Apply FTOPSIS to compare the overall performances of alternative suppliers.
Step 4. If experts’ authority levels are specified, go to Step 5; otherwise, derive the authority level of each expert based on the consistency of his/her judgment.
Step 5. Apply FWI to aggregate the comparison results by all experts.

**Figure 2:**
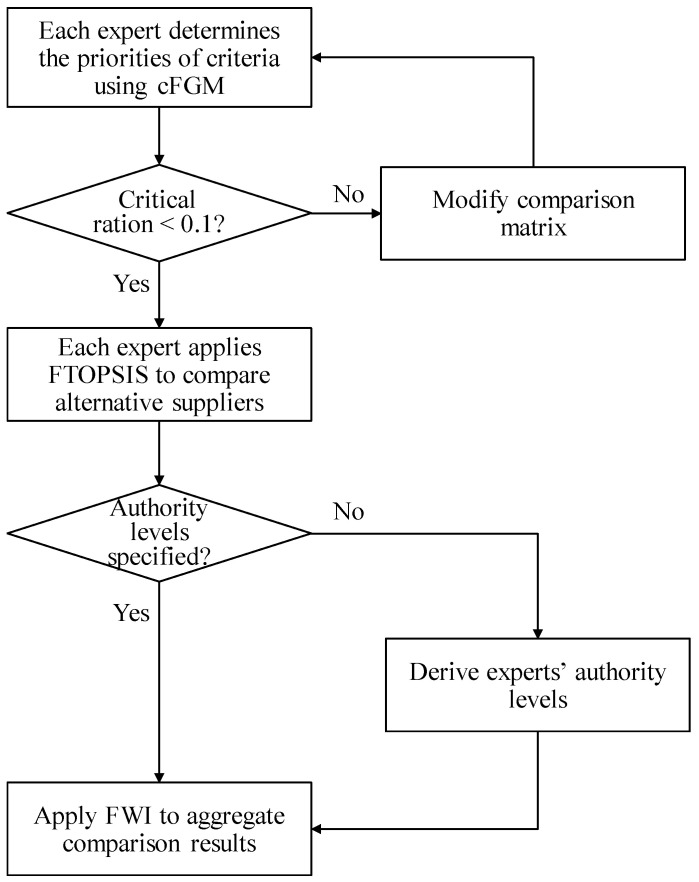
The steps of the proposed methodology.

A flowchart is provided in Figure 2 to show the steps of the proposed methodology.

### 3.1. Calibrated FGM Method for Determining the Priorities of Criteria

The fuzzy judgment matrix of expert *k* is denoted by $\tilde{A}(k)=[{\tilde{a}}_{ij}(k)]$; *k* = 1~*K*. ${\tilde{a}}_{ij}(k)$ indicates the relative priority of criterion *i* over criterion *j* to expert *k*. Then, the fuzzy eigenvalue $\tilde{\lambda }(k)$ and fuzzy vector $\tilde{x}(k)$ of $\tilde{A}(k)$ are derived as follows:

(1) $$\det(\tilde{A}(k)(-)\tilde{\lambda }(k)I)=0$$

(2) $$(\tilde{A}(k)(-)\tilde{\lambda }(k)I)(\times )\tilde{x}(k)=0$$

where (−) and (×) denote fuzzy subtraction and fuzzy multiplication, respectively [58]. The priorities of criteria $\tilde{w}(k)$ are obtained by normalizing $\tilde{x}(k)$:

(3) $${\tilde{w}}_{i}(k)=\frac{{\tilde{x}}_{i}(k)}{\sum_{l=1}^{n}{\tilde{x}}_{l}(k)}$$

When ${\tilde{a}}_{ij}(k)$ is represented by a triangular fuzzy number (TFN), $\tilde{\lambda }(k)$, $\tilde{x}(k)$ and ${\tilde{w}}_{i}(k)$ will not be TFNs anymore, because the multiplication of TFNs does not yield a TFN [58]. To derive the exact values of these fuzzy variables, enumeration-based methods, such as alpha-cut operations (ACO) [59] and approximating ACO (xACO) [26], are required. However, such enumeration-based techniques are time-consuming. To tackle this difficulty, FGM is commonly applied to approximate the values of ${\tilde{w}}_{i}(k)$ and $\tilde{\lambda }(k)$ with TFNs [19,20,21].

**Theorem 1** **[43].** $${\tilde{w}}_{i}(k)≅\frac{\sqrt[{n}]{\prod_{j=1}^{n}{\tilde{a}}_{ij}(k)}}{\sum_{l=1}^{n}\sqrt[{n}]{\prod_{j=1}^{n}{\tilde{a}}_{lj}(k)}}.$$

**Theorem** **2** **[43].** ${\tilde{w}}_{i}(k)≅(w_{i1}(k),w_{i2}(k),w_{i3}(k))$ *where*

(4) $$w_{i1}(k)=\frac{1}{1+\sum_{l\neq i}\frac{\sqrt[{n}]{\prod_{j=1}^{n}a_{lj3}(k)}}{\sqrt[{n}]{\prod_{j=1}^{n}a_{ij1}(k)}}}$$

(5) $$w_{i2}(k)=\frac{1}{1+\sum_{l\neq i}\frac{\sqrt[{n}]{\prod_{j=1}^{n}a_{lj2}(k)}}{\sqrt[{n}]{\prod_{j=1}^{n}a_{ij2}(k)}}}$$

(6) $$w_{i3}(k)=\frac{1}{1+\sum_{l\neq i}\frac{\sqrt[{n}]{\prod_{j=1}^{n}a_{lj1}(k)}}{\sqrt[{n}]{\prod_{j=1}^{n}a_{ij3}(k)}}}$$

**Theorem** **3** **[43].** ${\tilde{\lambda }}_{\max}(k)≅(\lambda_{\max,1}(k),\lambda_{\max,2}(k),\lambda_{\max,3}(k))$*where*

(7) $$\lambda_{\max,1}(k)=\max(n,1+\frac{1}{n}\sum_{i=1}^{n}\sum_{j\neq i}\frac{a_{ij1}(k)w_{j1}(k)}{w_{i3}(k)})$$

(8) $$\lambda_{\max,2}(k)=1+\frac{1}{n}\sum_{i=1}^{n}\sum_{j\neq i}\frac{a_{ij2}(k)w_{j2}(k)}{w_{i2}(k)}$$

(9) $$\lambda_{\max,3}(k)=1+\frac{1}{n}\sum_{i=1}^{n}\sum_{j\neq i}\frac{a_{ij3}(k)w_{j3}(k)}{w_{i1}(k)}$$

*The consistency of pairwise comparison results is evaluated in terms of fuzzy consistency ratio*$\tilde{CR}(k)$*as*

(10) $$\tilde{CR}(k)=\frac{\frac{{\tilde{\lambda }}_{\max}(k)-n}{n-1}}{RI}$$

*whereRI is random consistency index [60].*$\tilde{CR}(k)\geq 0$. $\tilde{CR}(k)$*needs to be less than 0.1, but can be relaxed to be less than 0.3 if the problem size is large [61]. Otherwise, the expert is asked to modify his/her pairwise comparison results.*

**Figure 3:**
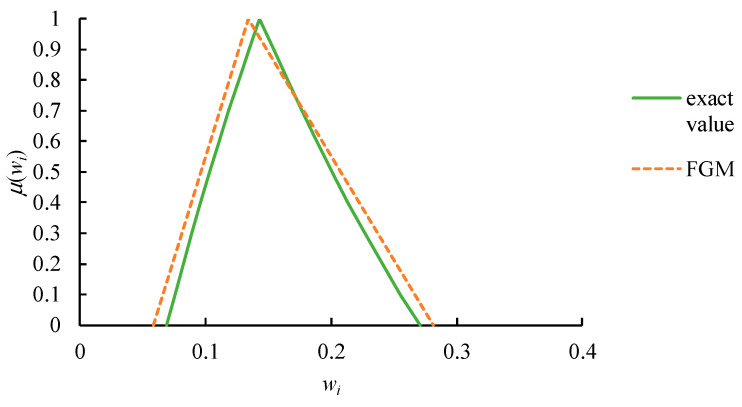
The inaccuracy of the approximation results using fuzzy geometric mean (FGM).

The approximation result using FGM is subject to inaccuracy, as illustrated in Figure 3. Obviously, $w_{i2}(k)$ deviates from its exact value, while $w_{i1}(k)$ and $w_{i3}(k)$ are less than and greater than their exact values, respectively.

The inaccuracy can be reduced by calibrating the approximation result towards the exact value as follows

(11) $$w_{i1}(k)\to w_{i1}(k)\cdot \max(\eta_{i2}(k),1/\eta_{i2}(k))$$

(12) $$w_{i2}(k)\to w_{i2}(k)\cdot \eta_{i2}(k)$$

(13) $$w_{i3}(k)\to w_{i3}(k)/\max(\eta_{i2}(k),1/\eta_{i2}(k))$$

where

(14) $$\eta_{i2}(k)=\frac{x_{ci}(k)}{\sum_{l=1}^{n}x_{cl}(k)}/w_{i2}(k)$$

and

(15) $$\det(A_{c}(k)-\lambda_{c}(k)I)=0$$

(16) $$(A_{c}(k)-\lambda_{c}(k)I)x_{c}(k)=0$$

**Figure 4:**
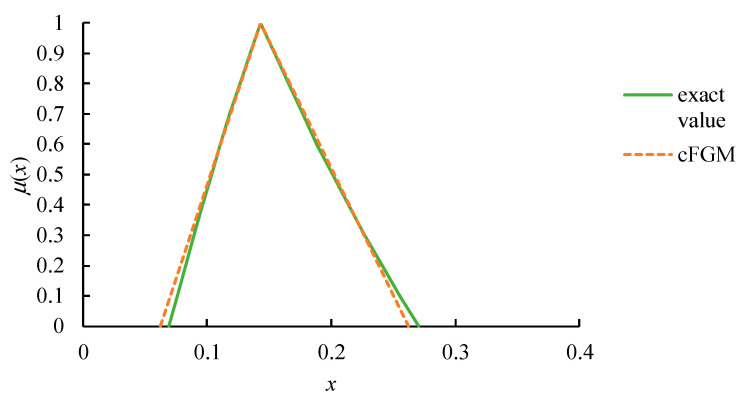
The calibration result.

$A_{c}(k)=[a_{ij2}(k)]$. Equations (15) and (16) are solved instantly by performing a single eigenanalysis by treating $\tilde{A}(k)$ as a crisp one. The calibration result is illustrated in Figure 4. 

### 3.2. FTOPSIS for Comparing Alternatives

Subsequently, FTOPSIS [8,9,14,22] is applied to compare the overall performances of alternative suppliers. First, the performance of an alternative supplier in optimizing each criterion is normalized using the fuzzy distributive normalization [62]:

(17) $$\begin{array}{cc} {\tilde{\rho }}_{qi} & =\frac{{\tilde{p}}_{qi}}{\sqrt{\sum_{\phi =1}^{Q}{\tilde{p}}_{\phi i}^{2}}}\\ & =\frac{1}{\sqrt{1+\sum_{\phi \neq q}{\left(\frac{{\tilde{p}}_{\phi i}}{{\tilde{p}}_{qi}}\right)}^{2}}} \end{array}$$

**Theorem** **4** **[22].** ${\tilde{\rho }}_{qi}≅(\rho_{qi1},\rho_{qi2},\rho_{qi3})$*where*

(18) $$\rho_{qi1}=\frac{1}{\sqrt{1+\sum_{\phi \neq q}{\left(\frac{p_{\phi i3}}{{\tilde{p}}_{qi1}}\right)}^{2}}}$$

(19) $$\rho_{qi2}=\frac{1}{\sqrt{1+\sum_{\phi \neq q}{\left(\frac{p_{\phi i2}}{{\tilde{p}}_{qi2}}\right)}^{2}}}$$

(20) $$\rho_{qi3}=\frac{1}{\sqrt{1+\sum_{\phi \neq q}{\left(\frac{p_{\phi i1}}{{\tilde{p}}_{qi3}}\right)}^{2}}}$$

*where*${\tilde{p}}_{qi}$*is the performance of the q-th alternative supplier in optimizing the i-th criterion;*${\tilde{\rho }}_{qi}$*is the normalized performance. Subsequently, the fuzzy weighted score is calculated based on the relative priorities derived using the cFGM method by each expert:*

(21) $${\tilde{s}}_{qi}(k)={\tilde{w}}_{i}(k)(\times ){\tilde{\rho }}_{qi}$$

Therefore, the same fuzzy weighted score will have *K* values.

After that, the fuzzy ideal (zenith) point and the fuzzy anti-ideal (nadir) point are specified, respectively, for each expert as

(22) $${\tilde{\Lambda }}^{+}(k)=\{{\tilde{\Lambda }}_{i}^{+}(k)\}=\{\underset{q}{\max}{\tilde{s}}_{qi}(k)\}$$

(23) $${\tilde{\Lambda }}^{-}(k)=\{{\tilde{\Lambda }}_{i}^{-}(k)\}=\{\underset{q}{\min}{\tilde{s}}_{qi}(k)\}$$

The fuzzy distance from each alternative supplier to the two points are calculated, respectively, as:

(24) $${\tilde{d}}_{q}^{+}(k)=\sqrt[{o(k)}]{\sum_{i=1}^{n}\max{\left({\tilde{\Lambda }}_{i}^{+}(k)(-){\tilde{s}}_{qi}(k),0\right)}^{o(k)}}$$

(25) $${\tilde{d}}_{q}^{-}(k)=\sqrt[{o(k)}]{\sum_{i=1}^{n}\max{\left({\tilde{s}}_{qi}(k)(-){\tilde{\Lambda }}_{i}^{-}(k),0\right)}^{o(k)}}$$

where o(k) reflects the sensitivity of expert *k* to any change in the overall performance of an alternative: from 1 (very sensitive) to 5 (very insensitive) [43]. In traditional FTOPSIS, o(k) is usually set to 2, i.e., the Euclidean distance. Finally, the fuzzy closeness of each alternative supplier is obtained as:

(26) $${\tilde{C}}_{q}(k)=\frac{{\tilde{d}}_{q}^{-}(k)}{{\tilde{d}}_{q}^{+}(k)(+){\tilde{d}}_{q}^{-}(k)}$$

**Figure 5:**
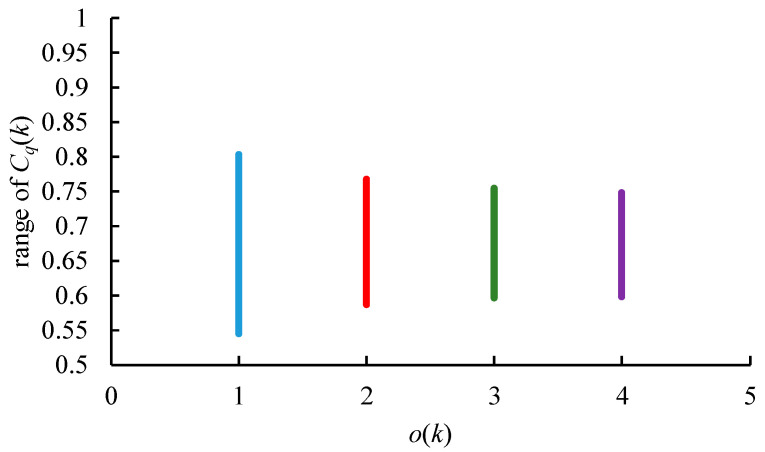
The effect of o(k) on ${\tilde{C}}_{q}(k)$.

$0\leq {\tilde{C}}_{q}(k)\leq 1$. An alternative is more suitable if its fuzzy closeness is higher. However, the assessment results by different experts are not equal, and need to be aggregated [46]. The effect of o(k) on the range of ${\tilde{C}}_{q}(k)$ is illustrated in Figure 5. As o(k) increases, the range of ${\tilde{C}}_{q}(k)$ shrinks, which means it becomes more difficult to discriminate the performances of alternative suppliers.

### 3.3. FWI for the Aggregation of the Comparison Results by All Experts

Most of the existing methods aggregate the pairwise comparison results by experts or the priorities of criteria derived by them [37]. Unlike the existing methods, in the proposed methodology, FWI [26] is applied to aggregate the comparison results by all experts as

(27) $${\tilde{C}}_{q}(all)=\tilde{FWI}(\{{\tilde{C}}_{q}(k)\})$$

with the following membership function:

(28) $$\mu_{{\tilde{C}}_{q}(all)}(x)=\underset{k}{\min}\mu_{{\tilde{C}}_{q}(k)}(x)+\sum_{k}(\omega_{k}-\underset{l}{\min}\omega_{l})(\mu_{{\tilde{C}}_{q}(k)}(x)-\underset{l}{\min}\mu_{{\tilde{C}}_{q}(l)}(x))$$

where $\omega_{k}$ is the authority level of expert *k*; $\sum_{k=1}^{K}\omega_{k}=1$. Some theoretical properties of the FWI operation are described below [26]:

(1) ${\tilde{C}}_{q}(all)={\tilde{C}}_{q}(l)$ if $\omega_{l}=1$ and $\omega_{k}=0$ ∀ *k* ≠ *l*
(2) ${\tilde{C}}_{q}(all)=\tilde{FI}(\{{\tilde{C}}_{q}(k)\})$ if $\omega_{k}=\frac{1}{K}$ ∀ *k*; $\tilde{FI}$ is the fuzzy intersection operator.
(3) $\underset{k}{\min}\mu_{{\tilde{C}}_{q}(k)}(x)\leq \mu_{{\tilde{C}}_{q}(all)}(x)\leq \underset{k}{\max}\mu_{{\tilde{C}}_{q}(k)}(x)$
(4) $\frac{\partial \mu_{{\tilde{C}}_{q}(all)}(x)}{\partial \mu_{{\tilde{C}}_{q}(k)}(x)}\propto \omega_{q}$

**Figure 6:**
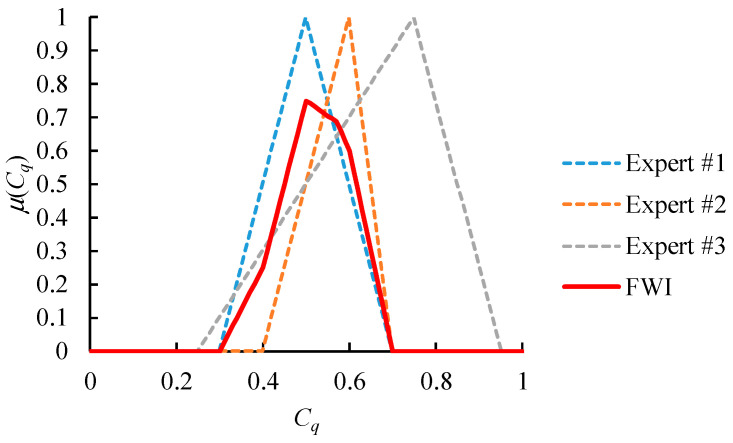
An example of the fuzzy weighted intersection (FWI) aggregator ({$\omega_{k}$} = (0.6, 0.3, 0.1)).

An example is provided in Figure 6. Values that are considered highly possible by all experts or just the most authoritative expert will have high memberships in the FWI result. This result is more in line with the expectations of all experts and is more acceptable to everyone.

If experts do not specify their unequal authority levels, then the authority level of each expert is automatically set to a value proportional to the consistency of his/her pairwise comparison results [43]:

(29) $$\omega_{k}=\frac{e^{-\frac{CR_{2}(k)}{0.1}}}{\sum_{l=1}^{K}e^{-\frac{CR_{2}(l)}{0.1}}}$$

There are other ways to determine the authority level of an expert. For example,

(30) $$\omega_{k}=\frac{\frac{1}{CR_{2}(k)}}{\sum_{l=1}^{K}\frac{1}{CR_{2}(l)}}$$

To get an absolute ranking, the fuzzy closeness of an alternative supplier can be defuzzified using the center-of-gravity (COG) method [63]:

(31) $$COG({\tilde{C}}_{q}(all))=\frac{\int_{0}^{1}x\mu_{{\tilde{C}}_{q}(all)}(x)dx}{\int_{0}^{1}\mu_{{\tilde{C}}_{q}(all)}(x)dx}$$

## 4. Application

### 4.1. Application of the Proposed Methodology

In this case, a wafer foundry in Taiwan would like to choose suitable alternative suppliers amid the COVID-19 pandemic. Current suppliers of the wafer foundry were mostly located in Taiwan, Netherlands, and the USA. Among these regions, the Netherlands and the USA have been highly impacted by the pandemic [64]. Therefore, looking for alternative suppliers of chemical mechanical planarization (CMP) slurries and CMP pads to replace the existing suppliers in the two regions became a critical task to the wafer foundry. To fulfill this task, the proposed methodology was applied.

The following factors were considered critical to the performance of an alternative supplier amid the COVID-19 pandemic:

- level of buyer–supplier cooperation [65,66],
- delivery speed [37,38,39,40],
- company reputation [37,38,39,40],
- pandemic containment performance [43], and
- pandemic severity [43].

Three experts were involved in the alternative supplier selection process. At first, each expert was requested to compare the relative priorities of criteria in pairs. However, whether there will be a vaccine for the COVID-19 pandemic at the time of supply was an issue that affected the judgment of experts. To address this issue, two scenarios were considered: 

Scenario I: Vaccines are already available.

Scenario II: There is no vaccine yet.

**Figure 7:**
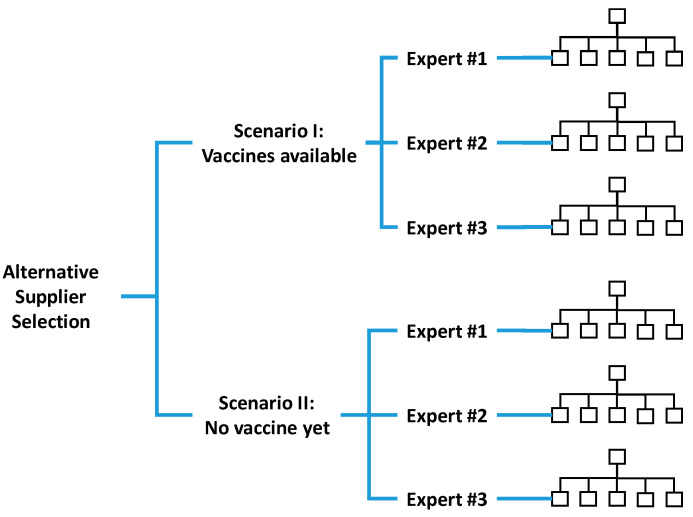
The scenario-based multi-criteria decision-making problem.

In fact, even if vaccines are available, the speed of obtaining vaccines varies from country to country. Different regions or ethnic groups in the same country also have different access times for vaccines. Nevertheless, since the availability of vaccines was considered in the two scenarios, “vaccine acquisition speed” was removed from the set of critical factors. This is why vaccine acquisition speed was not taken into account. Experts expressed their judgments for both scenarios. The scenario-based multi-criteria decision-making problem is illustrated in Figure 7.

The pairwise comparison results by the three experts in the two scenarios are summarized in Table 2.

The consistency of these fuzzy judgment matrixes were evaluated in terms of $\tilde{CR}(k)$. The results are presented in Table 3, which shows sufficient consistency for the subsequent operations.

**Figure 8:**
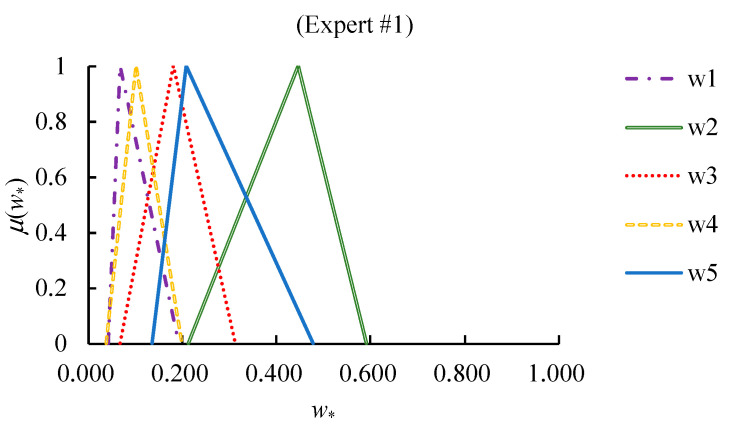

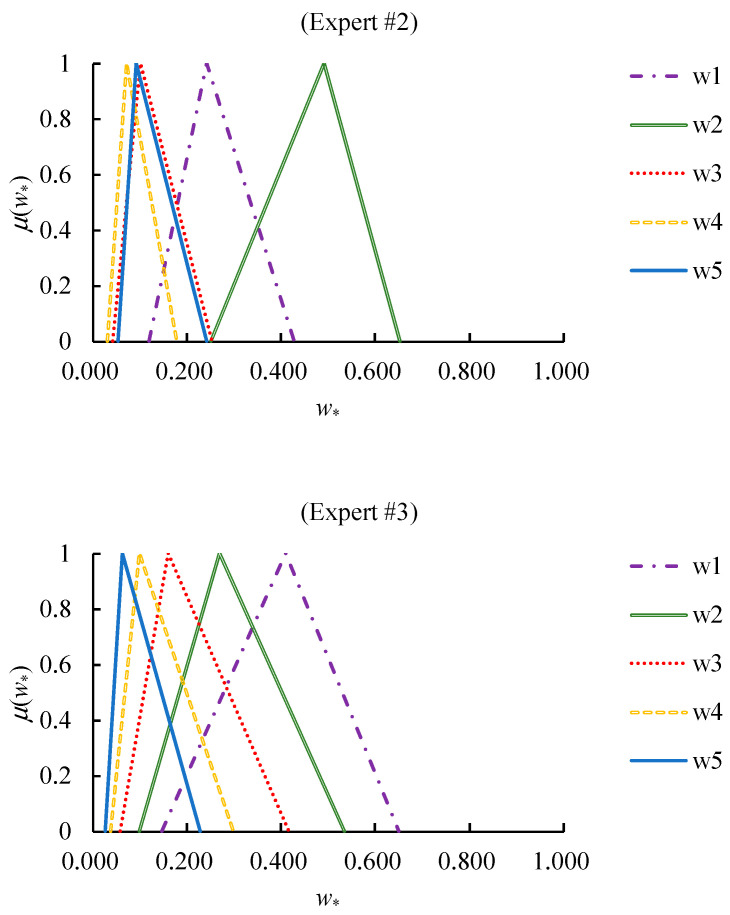
The values of fuzzy priorities in scenario I.

**Figure 9:**
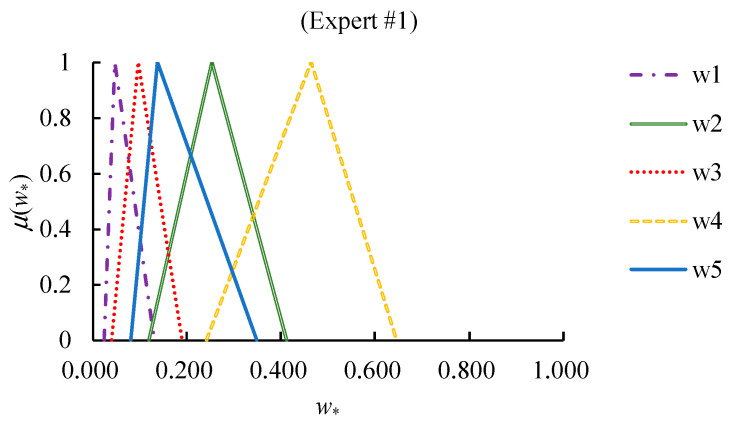

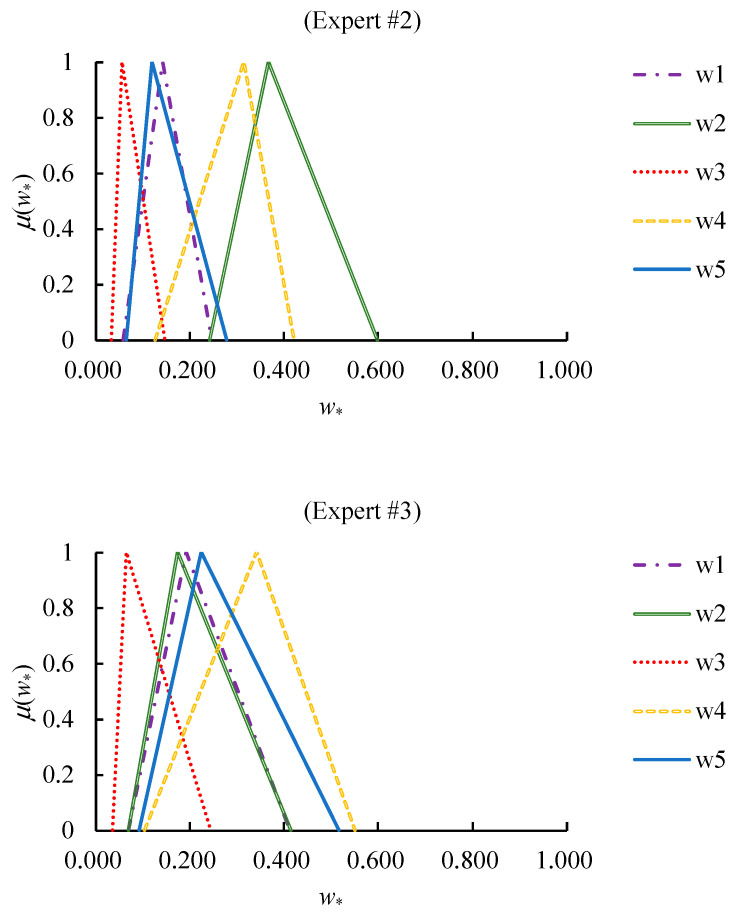
The values of fuzzy priorities in scenario II.

The cFGM method was applied by each expert to derive the values of priorities in both scenarios. The results are summarized in Figure 8 and Figure 9. 

Among the five criteria, “level of buyer–supplier cooperation” and “company reputation” were the-higher-the-better criteria, whereas the others were the-lower-the-better criteria. The performances in optimizing these criteria were evaluated according to the rules depicted in Table 4, based on the formulae proposed by [37,43].

The performances of three alternative suppliers in optimizing these criteria are summarized in Table 5.

FTOPSIS was applied by each expert to compare the overall performances of these alternative suppliers in both scenarios. Experts specified their sensitivities as

*o*(1) = 2

*o*(2) = 1

*o*(3) = 3

The comparison results are summarized in Table 6. 

Obviously, the comparison results by different experts were not equal, and needed to be aggregated. To this end, FWI was applied. The authority levels of these experts were determined according to their consistency ratios (see Table 7).

**Figure 10:**
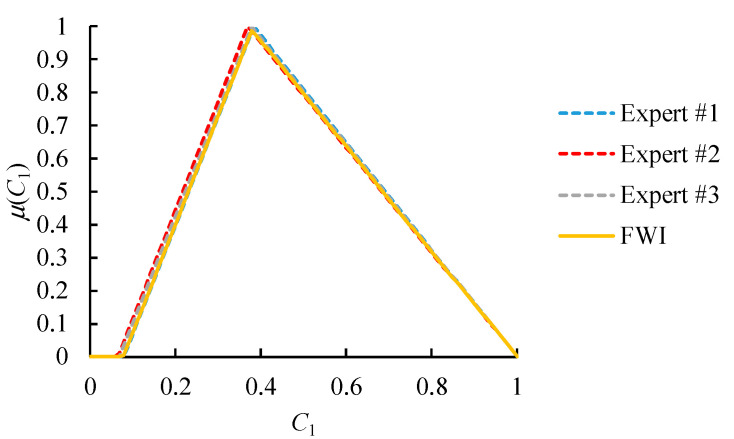

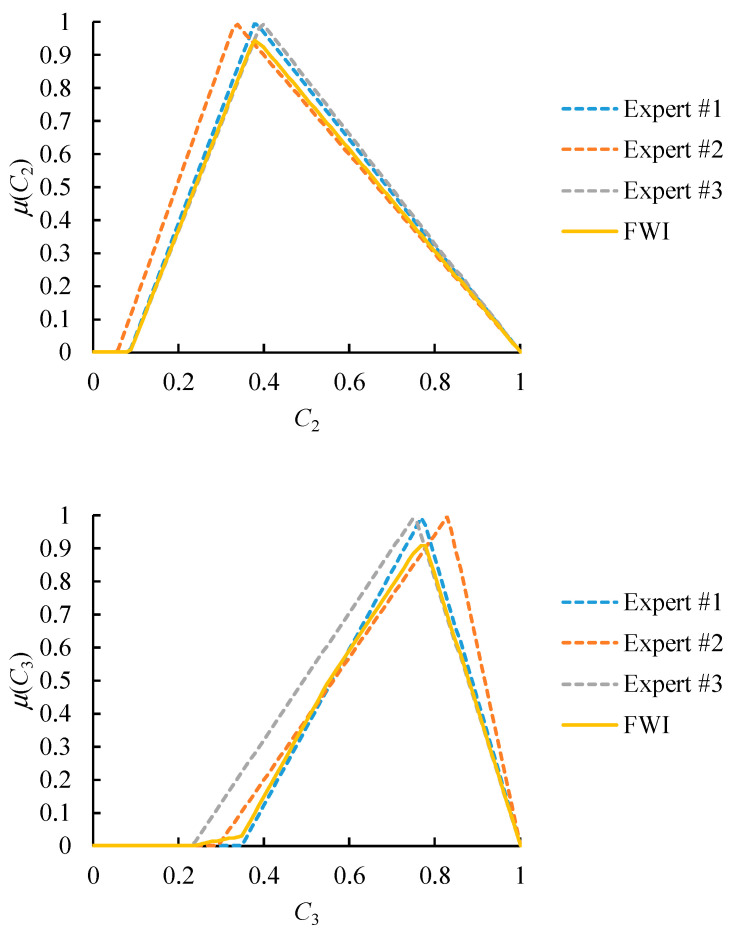
The aggregation results (scenario I).

**Figure 11:**
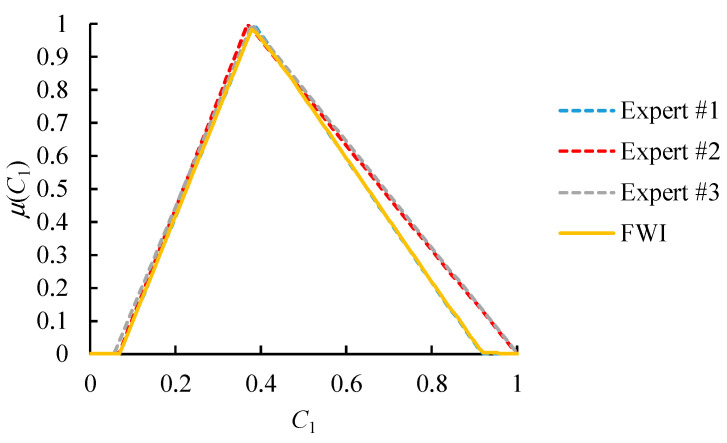

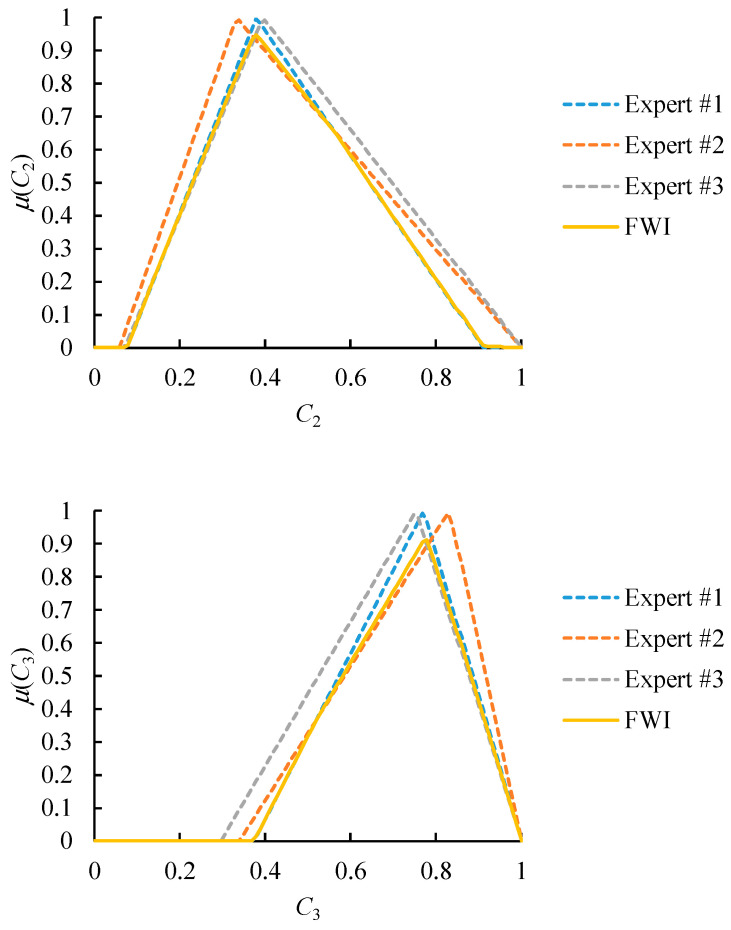
The aggregation results (scenario II).

The aggregation results are shown in Figure 10 and Figure 11. After defuzzifying the aggregation results using COG, as shown in Table 8, the sequence of these alternative suppliers was obtained.

### 4.2. Discussion

According to the experimental results, the following discussion is made:

(1) When vaccines for the COVID-19 pandemic were expected to emerge, experts believed that “delivery speed” and “level of buyer–supplier cooperation” were more important criteria than the others. In contrast, without COVID-19 vaccines, “pandemic containment performance” and “delivery speed” were considered to be the first two important criteria.
(2) As expected, the pairwise comparison results by experts in different scenarios varied greatly.
(3) The overall performances of alternative suppliers, in terms of their closenesses, evaluated by different experts were quite similar
(4) The difference between the two scenarios did affect the decisions of experts. For example, Expert #1 thought that Alternative Supplier #2 was better than Alternative Supplier #1 in Scenario I, but preferred Alternative Supplier #1 to Alternative Supplier #2 in Scenario II.
(5) The comparison results also showed that no matter which scenario was considered, Alternative Supplier #3 was always the best choice. Therefore, this choice was quite robust.
(6) For comparison, two existing methods were also applied to this case. The first method was the FGM-FGM-fuzzy weighted average (FWA) method, in which FGM was applied to aggregate experts’ fuzzy judgment matrixes and to derive the priorities of criteria from the aggregation result. Subsequently, FWA was applied to evaluate the overall performance of each alternative supplier. The second method was the FGM-FEA-FWA method, in which FEA was applied to derive the priorities of criteria instead. The ranking results using various methods are compared in Table 9.
(7) It is interesting to know whether the consideration of different criteria changes the comparison result. In order to investigate this issue, an experiment was conducted by dropping one of the five criteria at a time and alternative suppliers were compared based on the remaining criteria. The experimental results are summarized in Table 10. Alternative Supplier #3 was always the best choice. In addition, the ranking results in the two scenarios differed when “pandemic containment performance” or “pandemic severity” was removed.
(8) One contribution of this research is that issues related to the COVID-19 pandemic were considered in the selection of alternative suppliers, which has not yet been fully resolved. On the contrary, past studies have reported the disruption of supply chains by the COVID-19 pandemic [34,35,46], identified and assessed the risks faced by organizations [34], identified factors or barriers to the sustainability of an organization amid the COVID-19 pandemic [36,43,61], or discussed treatments (including contract management [35], workforce management [35], and demand management [46]) that could be taken to mitigate the impact. Biswas et al. [68] also applied a FAHP method for a similar purpose amid the COVID-19 pandemic. However, their FAHP method was based on the compromise among all experts, while the cFGM-FTOPSIS-FWI approach proposed in this study sought the consensus among all experts.
(9) In the view of Chen et al. [43], pandemic containment performance, pandemic severity, vaccine acquisition speed, demand shrinkage, supplier impact, and infection risk affect the robustness of a factory to the COVID-19 pandemic. A supplier faces the same risks and can take similar measures (e.g., wearing masks, physical distancing, moving raw material inventory to places free from quarantine and easy to ship, securing future transportation services, negotiating with customers on possible delays or cancellation, etc.) to mitigate the impact [4,6,43]. In addition, compared with downstream assemblers, upstream raw material suppliers have a lower degree of automation, so they may be more susceptible to these risks. However, due to the COVID-19 pandemic, some suppliers have shut down, which is an opportunity for other suppliers because they can increase their prices.
(10) If the results of the two scenarios were different, the wafer foundry could choose the best alternative suppliers of the two scenarios and allocate the required quantity of raw materials between the two alternative suppliers.

## 5. Conclusions

The supply chain disruption caused by the COVID-19 pandemic has forced manufacturers to look for alternative suppliers. However, how to choose a suitable alternative supplier in the COVID-19 pandemic has rarely been investigated. In order to fill this gap, this research proposes a cFGM-FTOPSIS-FWI approach. In the proposed methodology, first, the cFGM method was proposed to improve the accuracy of deriving the priorities of criteria, which are essential for selecting a suitable alternative supplier. Subsequently, each expert applied the FTOPSIS method to compare the overall performances of alternative suppliers in the COVID-19 pandemic. The sensitivity of an expert to any change in the overall performance of an alternative supplier was also considered. Finally, the FWI operator was applied to aggregate the comparison results by all experts. For this purpose, an expert’s authority level was set to a value proportional to the consistency of his/her pairwise comparison results.

The cFGM-FTOPSIS-FWI approach was applied to select suitable alternative suppliers for a Taiwanese foundry in the COVID-19 pandemic. Two scenarios were considered. In order to elaborate on the effectiveness of the cFGM-FTOPSIS-FWI approach, two existing methods were also applied to this case. Finally, the following conclusions were drawn from the experimental results:

(1) In the absence of a COVID-19 vaccine, “pandemic containment performance” was considered the most important criterion. On the contrary, if vaccines will be available, “delivery speed” was the highest priority.
(2) Experts have made different decisions in different scenarios.
(3) However, after aggregation, Alternative Supplier #3 was always the best choice regardless of the considered scenario.
(4) The result of alternative supplier selection using the proposed methodology was the same as those using two existing methods, showing the robustness of the proposed methodology.
(5) If more experts are involved, or if more alternative suppliers are considered, the selection result will be different.

The proposed methodology also has limitations. For example, the performance of an alternative supplier in optimizing certain criteria, such as delivery speed, pandemic containment performance, and pandemic severity, may fluctuate significantly, which can cause difficulties in making a longer-term decision. In addition, the consistency of an expert’s judgment may not well reflect his/her level of authority in making a joint decision.

In this study, a wafer foundry in Taiwan was studied. The proposed methodology can also be applied to other sectors or industries in countries or regions with different government policies and structures. In addition, the proposed methodology can be extended to handle situations where experts lack overall consensus. Further, there is considerable uncertainty in the delivery of COVID-19 vaccines. Fuzzy or stochastic models that take this into account can be adopted to make more precise decisions. These constitute some directions for future research.

## Figures and Tables

**Table 1:** Differences between the proposed methodology and some existing methods.

Method	Expert Inputs	Expert’s Authority Levels	How Authority Levels Are Derived	Method for Deriving Priorities	Aggregation Method
Zheng et al. [12]	Relative priorities of criteria	Equal	-	FGM	Discussion
Chen [20]	Relative priorities of criteria	Equal	-	FGM	FGM
Chen et al. [26]	Forecast	Unequal	Subjectively assigned	-	FWI
Gao et al. [27]	Relative priorities of criteria	Equal	-	FGM	FGM
Wang et al. [28]	Relative priorities of criteria	Equal	-	FEA	FGM
Lin et al. [29]	Relative priorities of criteria	Equal	-	FI	FGM
The proposed methodology	Relative priorities of criteria Expert’s sensitivity	Unequal	Automatically assigned	cFGM	FWI

FGM (fuzzy geometric mean); FWI (fuzzy weighted intersection); FEA (uzzy extent analysis); FI (fuzzy intersection); cFGM (calibrated fuzzy geometric mean).

**Table 2:** Summary of pairwise comparison results in the two scenarios.

Expert #	Scenario I	Scenario II
1	$\left[\begin{array}{ccccc} 1 & - & - & \left(1,1,3\right) & -\\ \left(5,7,9\right) & 1 & \left(3,5,7\right) & \left(2,4,6\right) & -\\ \left(1,3,5\right) & - & 1 & \left(1,3,5\right) & -\\ - & - & - & 1 & -\\ \left(1,3,5\right) & \left(1,1,3\right) & \left(1,1,3\right) & \left(1,1,3\right) & 1 \end{array}\right]$	$\left[\begin{array}{ccccc} 1 & - & - & - & -\\ \left(5,7,9\right) & 1 & \left(3,5,7\right) & - & -\\ \left(1,3,5\right) & - & 1 & - & -\\ \left(3,5,7\right) & \left(1,3,5\right) & \left(3,5,7\right) & 1 & \left(2,4,6\right)\\ \left(1,3,5\right) & \left(1,1,3\right) & \left(1,1,3\right) & - & 1 \end{array}\right]$
2	$\left[\begin{array}{ccccc} 1 & - & \left(1,3,5\right) & \left(3,5,7\right) & \left(2,4,6\right)\\ \left(3,5,7\right) & 1 & \left(2,4,6\right) & \left(1,3,5\right) & \left(3,5,7\right)\\ - & - & 1 & \left(1,3,5\right) & -\\ - & - & - & 1 & -\\ - & - & \left(1,2,4\right) & \left(1,1,3\right) & 1 \end{array}\right]$	$\left[\begin{array}{ccccc} 1 & - & \left(1,3,5\right) & - & \left(1,3,5\right)\\ \left(3,5,7\right) & 1 & \left(2,4,6\right) & \left(1,1,3\right) & \left(2,4,6\right)\\ - & - & 1 & - & -\\ \left(3,5,7\right) & - & \left(3,5,7\right) & 1 & -\\ - & - & \left(1,2,4\right) & \left(1,1,3\right) & 1 \end{array}\right]$
3	$\left[\begin{array}{ccccc} 1 & \left(1,3,5\right) & \left(1,3,5\right) & \left(1,3,5\right) & \left(2,4,6\right)\\ - & 1 & \left(1,3,5\right) & \left(2,4,6\right) & \left(1,3,5\right)\\ - & - & 1 & \left(1,3,5\right) & \left(1,3,5\right)\\ - & - & - & 1 & \left(1,3,5\right)\\ - & - & - & - & 1 \end{array}\right]$	$\left[\begin{array}{ccccc} 1 & \left(1,3,5\right) & \left(1,3,5\right) & - & -\\ - & 1 & \left(1,3,5\right) & \left(1,1,3\right) & -\\ - & - & 1 & - & -\\ \left(1,3,5\right) & - & \left(1,3,5\right) & 1 & \left(1,3,5\right)\\ \left(1,3,5\right) & \left(1,1,3\right) & \left(1,3,5\right) & - & 1 \end{array}\right]$

**Table 3:** Consistency evaluation results.

*k*	Scenario #1	Scenario #2
1	(0.00,0.10,6.99)	(0.00,0.09,6.42)
2	(0.00,0.17,8.70)	(0.00,0.15,7.30)
3	(0.00,0.12,14.17)	(0.00,0.16,13.25)

**Table 4:** Rules for evaluating the performances in optimizing critical factors.

Criterion	Rule
Level of buyer–supplier cooperation	${\tilde{p}}_{q1}(x_{q})=\{\begin{array}{ccc} \left(0,0,1\right) & \mathrm{if} & x_{q}=\mathrm{very}\;\mathrm{low}\\ \left(0,1,2\right) & \mathrm{if} & x_{q}=\mathrm{low}\\ \left(1.5,2.5,3.5\right) & \mathrm{if} & x_{q}=\mathrm{moderate}\\ \left(3,4,5\right) & \mathrm{if} & x_{q}=\mathrm{high}\\ \left(4,5,5\right) & \mathrm{if} & x_{q}=\mathrm{very}\;\mathrm{high} \end{array}$ where $x_{q}$ is the level of buyer–supplier cooperation.
Delivery speed	${\tilde{p}}_{q2}(x_{q})=\{\begin{array}{ccc} \left(0,0,1\right) & \mathrm{if} & x_{q}\leq 0.9\cdot \underset{r}{\min}x_{r}+0.1\cdot \underset{r}{\max}x_{r}\;\mathrm{or}\;\mathrm{data}\;\mathrm{not}\;\mathrm{available}\\ \left(0,1,2\right) & \mathrm{if} & 0.9\cdot \underset{r}{\min}x_{r}+0.1\cdot \underset{r}{\max}x_{r}\leq x_{q}<0.65\cdot \underset{r}{\min}x_{r}+0.35\cdot \underset{r}{\max}x_{r}\\ \left(1.5,2.5,3.5\right) & \mathrm{if} & 0.65\cdot \underset{r}{\min}x_{r}+0.35\cdot \underset{r}{\max}x_{r}\leq x_{q}<0.35\cdot \underset{r}{\min}x_{r}+0.65\cdot \underset{r}{\max}x_{r}\\ \left(3,4,5\right) & \mathrm{if} & 0.35\cdot \underset{r}{\min}x_{r}+0.65\cdot \underset{r}{\max}x_{r}\leq x_{q}<0.1\cdot \underset{r}{\min}x_{r}+0.9\cdot \underset{r}{\max}x_{r}\\ \left(4,5,5\right) & \mathrm{if} & 0.1\cdot \underset{r}{\min}x_{r}+0.9\cdot \underset{r}{\max}x_{r}<x_{q} \end{array}$ where $x_{q}$ is the average delivery time.
Company reputation	${\tilde{p}}_{q3}(x_{q})=\{\begin{array}{ccc} \left(0,0,1\right) & \mathrm{if} & x_{q}=\mathrm{very}\;\mathrm{poor}\\ \left(0,1,2\right) & \mathrm{if} & x_{q}=\mathrm{poor}\\ \left(1.5,2.5,3.5\right) & \mathrm{if} & x_{q}=\mathrm{moderate}\\ \left(3,4,5\right) & \mathrm{if} & x_{q}=\mathrm{good}\\ \left(4,5,5\right) & \mathrm{if} & x_{q}=\mathrm{very}\;\mathrm{good} \end{array}$ where $x_{q}$ is the company reputation of the alternative supplier.
Pandemic containment performance	${\tilde{p}}_{q4}(x_{q})=\{\begin{array}{ccc} \left(0,0,1\right) & \mathrm{if} & x_{q}<0.9\cdot \underset{r}{\min}x_{r}+0.1\cdot \underset{r}{\max}x_{r}\mathrm{or}\;\mathrm{data}\;\mathrm{not}\;\mathrm{available}\\ \left(0,1,2\right) & \mathrm{if} & 0.9\cdot \underset{r}{\min}x_{r}+0.1\cdot \underset{r}{\max}x_{r}\leq x_{q}<0.65\cdot \underset{r}{\min}x_{r}+0.35\cdot \underset{r}{\max}x_{r}\\ \left(1.5,2.5,3.5\right) & \mathrm{if} & 0.65\cdot \underset{r}{\min}x_{r}+0.35\cdot \underset{r}{\max}x_{r}\leq x_{q}<0.35\cdot \underset{r}{\min}x_{r}+0.65\cdot \underset{r}{\max}x_{r}\\ \left(3,4,5\right) & \mathrm{if} & 0.35\cdot \underset{r}{\min}x_{r}+0.65\cdot \underset{r}{\max}x_{r}\leq x_{q}<0.1\cdot \underset{r}{\min}x_{r}+0.9\cdot \underset{r}{\max}x_{r}\\ \left(4,5,5\right) & \mathrm{if} & 0.1\cdot \underset{r}{\min}x_{r}+0.9\cdot \underset{r}{\max}x_{r}\leq x_{q} \end{array}$ where $x_{q}$ is the recovery index of the region [64].
Pandemic severity	${\tilde{p}}_{q5}(x_{k})=\{\begin{array}{ccc} \left(0,0,1\right) & \mathrm{if} & 0.1\cdot \underset{r}{\min}x_{r}+0.9\cdot \underset{r}{\max}x_{r}\leq x_{q}\;\mathrm{or}\;\mathrm{data}\;\mathrm{not}\;\mathrm{available}\\ \left(0,1,2\right) & \mathrm{if} & 0.35\cdot \underset{r}{\min}x_{r}+0.65\cdot \underset{r}{\max}x_{r}\leq x_{q}<0.1\cdot \underset{r}{\min}x_{r}+0.9\cdot \underset{r}{\max}x_{r}\\ \left(1.5,2.5,3.5\right) & \mathrm{if} & 0.65\cdot \underset{r}{\min}x_{r}+0.35\cdot \underset{r}{\max}x_{r}\leq x_{q}<0.35\cdot \underset{r}{\min}x_{r}+0.65\cdot \underset{r}{\max}x_{r}\\ \left(3,4,5\right) & \mathrm{if} & 0.9\cdot \underset{r}{\min}x_{r}+0.1\cdot \underset{r}{\max}x_{r}\leq x_{q}<0.65\cdot \underset{r}{\min}x_{r}+0.35\cdot \underset{r}{\max}x_{r}\\ \left(4,5,5\right) & \mathrm{if} & x_{q}<0.9\cdot \underset{r}{\min}x_{r}+0.1\cdot \underset{r}{\max}x_{r} \end{array}$ where $x_{q}$ is the current number of active cases in the region [67].

**Table 5:** Performance of the three alternative suppliers in optimizing various criteria.

*q*	1	2	3
${\tilde{p}}_{q1}$	(3, 4, 5)	(4, 5, 5)	(1.5, 2.5, 3.5)
${\tilde{p}}_{q2}$	(1.5, 2.5, 3.5)	(0, 0, 1)	(4, 5, 5)
${\tilde{p}}_{q3}$	(3, 4, 5)	(4, 5, 5)	(1.5, 2.5, 3.5)
${\tilde{p}}_{q4}$	(0, 0, 1)	(0, 0, 1)	(4, 5, 5)
${\tilde{p}}_{q5}$	(0, 0, 1)	(0, 0, 1)	(4, 5, 5)

**Table 6:** Comparison results.

	Scenario I				Scenario II			
Expert #1	*q*	${\tilde{C}}_{q}$	$COG({\tilde{C}}_{q})$	Rank	*q*	${\tilde{C}}_{q}$	$COG({\tilde{C}}_{q})$	Rank
	1	(0.081, 0.385, 1.000)	0.489	3	1	(0.071, 0.385, 0.916)	0.457	2
	2	(0.087, 0.381, 1.000)	0.489	2	2	(0.078, 0.381, 0.911)	0.457	3
	3	(0.350, 0.773, 1.000)	0.801	1	3	(0.376, 0.773, 1.000)	0.716	1
Expert #2	1	(0.067, 0.370, 1.000)	0.479	2	1	(0.069, 0.370, 0.999)	0.479	2
	2	(0.058, 0.334, 1.000)	0.464	3	2	(0.060, 0.334, 0.999)	0.464	3
	3	(0.295, 0.833, 1.000)	0.709	1	3	(0.341, 0.833, 1.000)	0.725	1
Expert #3	1	(0.070, 0.380, 1.000)	0.483	3	1	(0.058, 0.380, 1.000)	0.479	3
	2	(0.088, 0.396, 1.000)	0.495	2	2	(0.072, 0.396, 1.000)	0.490	2
	3	(0.234, 0.755, 1.000)	0.663	1	3	(0.298, 0.755, 1.000)	0.684	1

**Table 7:** Experts’ authority levels.

	Scenario I	Scenario II
Expert #1	0.40	0.50
Expert #2	0.24	0.26
Expert #3	0.37	0.24

**Table 8:** Defuzzification results.

*q*	Scenario I		Scenario II	
	$COG({\tilde{C}}_{q}(all))$	Rank	$COG({\tilde{C}}_{q}(all))$	Rank
1	0.4868	3	0.4580	3
2	0.4894	2	0.4584	2
3	0.6981	1	0.7148	1

**Table 9:** Comparison of ranking results using various methods (Scenario II).

Method	Ranking Result
FGM-FGM-FWA	3 → 1 → 2
FGM-FEA-FWA	3 → 1 → 2
The proposed methodology	3 → 2 → 1

FGM (fuzzy geometric mean); FWA (fuzzy weighted average); FEA (uzzy extent analysis).

**Table 10:** The ranking result after dropping a criterion at a time.

Considered Criteria	Scenario I	Scenario II
Delivery speed, company reputation, pandemic containment performance, pandemic severity	3 → 2 → 1	3 → 2 → 1
Level of buyer–supplier cooperation, company reputation, pandemic containment performance, pandemic severity	3 → 1 → 2	3 → 1 → 2
Level of buyer–supplier cooperation, delivery speed, pandemic containment performance, pandemic severity	3 → 2 → 1	3 → 2 → 1
Level of buyer–supplier cooperation, delivery speed, company reputation, pandemic severity	3 → 2 → 1	3 → 1 → 2
Level of buyer–supplier cooperation, delivery speed, company reputation, pandemic containment performance	3 → 2 → 1	3 → 1 → 2

## Data Availability

Data is contained within the article.

## References

[1] Wilgress-Pipe S. Coronavirus: Rolls-Royce Announces Factory Shutdown. https://www.thenationalnews.com/lifestyle/motoring/coronavirus-rolls-royce-announces-factory-shutdown-1.994340.

[2] Food Dive Team Tracking Coronavirus Closures at Food and Beverage Factories. https://www.fooddive.com/news/tracking-coronavirus-closures-at-food-and-beverage-factories/576559/.

[3] Eisenstein P.A. GM Cuts Production at Two Plants as Pandemic Squeezes Supply Chain. https://www.nbcnews.com/business/autos/gm-cuts-production-two-plants-coronavirus-pandemic-squeezes-supply-chain-n1247742.

[4] Kilpatrick J. COVID-19: Managing Supply Chain Risk and Disruption. https://www2.deloitte.com/global/en/pages/risk/articles/covid-19-managing-supply-chain-risk-and-disruption.html.

[5] Howard P.W. 2021 Ford Bronco Deliveries Delayed Until Summer Because of COVID-19 Supply Chain Disruptions. https://www.usatoday.com/story/money/cars/2020/12/05/2021-ford-bronco-coronavirus-delays-summer/3840894001/.

[6] Keegan K. COVID-19: Operations and Supply Chain Disruption. https://www.pwc.com/us/en/library/covid-19/supply-chain.html.

[7] Supply and Demand Chain Executive, Companies Pursue Alternative Suppliers to Spread Supply Chain Risks and Build Resilience to Mitigate COVID-19 Impact. https://www.sdcexec.com/risk-compliance/press-release/21203708/companies-pursue-alternative-suppliers-to-spread-supply-chain-risks-and-build-resilience-to-mitigate-covid19-impact.

[8] Boran F.E., Genç S., Kurt M., Akay D. (2009). A multi-criteria intuitionistic fuzzy group decision making for supplier selection with TOPSIS method. Expert Syst. Appl..

[9] Chen Z., Yang W. (2011). An MAGDM based on constrained FAHP and FTOPSIS and its application to supplier selection. Math. Comput. Model..

[10] Wu H.C., Wang Y.C., Chen T.C.T. (2020). Assessing and comparing COVID-19 intervention strategies using a varying partial consensus fuzzy collaborative intelligence approach. Mathematics.

[11] Dubois D., Prade H. (2012). Gradualness, uncertainty and bipolarity: Making sense of fuzzy sets. Fuzzy Sets Syst..

[12] Zheng G., Zhu N., Tian Z., Chen Y., Sun B. (2012). Application of a trapezoidal fuzzy AHP method for work safety evaluation and early warning rating of hot and humid environments. Saf. Sci..

[13] Tavana M., Zareinejad M., Di Caprio D., Kaviani M.A. (2016). An integrated intuitionistic fuzzy AHP and SWOT method for outsourcing reverse logistics. Appl. Soft Comput..

[14] Cevik Onar S., Oztaysi B., Kahraman C. (2014). Strategic decision selection using hesitant fuzzy TOPSIS and interval type-2 fuzzy AHP: A case study. Int. J. Comput. Intell. Syst..

[15] Acar C., Beskese A., Temur G.T. (2018). Sustainability analysis of different hydrogen production options using hesitant fuzzy AHP. Int. J. Hydrogen Energy.

[16] Albahri A.S., Al-Obaidi J.R., Zaidan A.A., Albahri O.S., Hamid R.A., Zaidan B.B., Alamoodi A.H., Hashim M. (2020). Multi-biological laboratory examination framework for the prioritization of patients with COVID-19 based on integrated AHP and group VIKOR methods. Int. J. Inf. Technol. Decis. Mak..

[17] Chen T., Lin C.-W. (2020). Smart and automation technologies for ensuring the long-term operation of a factory amid the COVID-19 pandemic: An evolving fuzzy assessment approach. Int. J. Adv. Manuf. Technol..

[18] Panetta K. A Framework for Executive Decision Making During COVID-19. https://www.gartner.com/smarterwithgartner/a-framework-for-executive-decision-making-during-covid-19/.

[19] Pan N.F. (2008). Fuzzy AHP approach for selecting the suitable bridge construction method. Autom. Constr..

[20] Chen T. (2020). Assessing factors critical to smart technology applications in mobile health care—The FGM-FAHP approach. Health Policy Technol..

[21] Wu H.-C., Chen T.-C.T., Huang C.-H., Shi Y.-C. (2020). Comparing built-in power banks for a smart backpack design using an auto-weighting fuzzy-weighted-intersection FAHP approach. Mathematics.

[22] Chen T.C.T., Lin Y.C. (2020). A FAHP-FTOPSIS approach for bioprinter selection. Health Technol..

[23] Chang D.Y. (1996). Applications of the extent analysis method on fuzzy AHP. Eur. J. Oper. Res..

[24] Chen T.C.T., Honda K. (2019). Fuzzy Collaborative Forecasting and Clustering: Methodology, System Architecture, and Applications.

[25] Chen T., Lin Y.C. (2008). A fuzzy-neural system incorporating unequally important expert opinions for semiconductor yield forecasting. Int. J. Uncertain. Fuzziness Knowl. Based Syst..

[26] Chen T.C.T., Wang Y.C., Lin C.W. (2020). A fuzzy collaborative forecasting approach considering experts’ unequal levels of authority. Appl. Soft Comput..

[27] Gao H., Ju Y., Gonzalez E.D.S., Zhang W. (2019). Green supplier selection in electronics manufacturing: An approach based on consensus decision making. J. Clean. Prod..

[28] Wang Y.C., Chen T., Yeh Y.L. (2019). Advanced 3D printing technologies for the aircraft industry: A fuzzy systematic approach for assessing the critical factors. Int. J. Adv. Manuf. Technol..

[29] Lin Y.C., Wang Y.C., Chen T.C.T., Lin H.F. (2019). Evaluating the suitability of a smart technology application for fall detection using a fuzzy collaborative intelligence approach. Mathematics.

[30] Weissman R. Navigating Supplier Relationships in the COVID-19 Era. https://www.supplychaindive.com/news/coronavirus-supplier-contracts-relationships/576132/.

[31] Howorth D. Will COVID-19 Spark a New Approach to Retailer/Supplier Relationships?. https://www.foodmanufacture.co.uk/Article/2020/11/13/Will-COVID-19-spark-a-new-approach-to-retailer-supplier-relationships.

[32] Ivanov D. (2020). Predicting the impacts of epidemic outbreaks on global supply chains: A simulation-based analysis on the coronavirus outbreak (COVID-19/SARS-CoV-2) case. Transp. Res. E Logist. Transp. Rev..

[33] Hoek R.V. (2020). Responding to COVID-19 supply chain risks—Insights from supply chain change management, total cost of ownership and supplier segmentation theory. Logistics.

[34] Sharma M., Luthra S., Joshi S., Kumar A. (2020). Developing a framework for enhancing survivability of sustainable supply chains during and post-COVID-19 pandemic. Int. J. Logist. Res. Appl..

[35] Majumdar A., Shaw M., Sinha S.K. (2020). COVID-19 debunks the myth of socially sustainable supply chain: A case of the clothing industry in South Asian countries. Sustain. Prod. Consum..

[36] Ivanov D., Dolgui A. (2020). Viability of intertwined supply networks: Extending the supply chain resilience angles towards survivability. A position paper motivated by COVID-19 outbreak. Int. J. Prod. Res..

[37] Wang Y.C., Chen T.C.T. (2019). A partial-consensus posterior-aggregation FAHP method—Supplier selection problem as an example. Mathematics.

[38] Yazdani M., Chatterjee P., Zavadskas E.K., Zolfani S.H. (2017). Integrated QFD-MCDM framework for green supplier selection. J. Clean. Prod..

[39] Alikhani R., Torabi S.A., Altay N. (2019). Strategic supplier selection under sustainability and risk criteria. Int. J. Prod. Econ..

[40] Hosseini S., Morshedlou N., Ivanov D., Sarder M.D., Barker K., Al Khaled A. (2019). Resilient supplier selection and optimal order allocation under disruption risks. Int. J. Prod. Econ..

[41] Mani V., Gunasekaran A., Delgado C. (2018). Enhancing supply chain performance through supplier social sustainability: An emerging economy perspective. Int. J. Prod. Econ..

[42] Niu B., Li J., Zhang J., Cheng H.K., Tan Y. (2019). Strategic analysis of dual sourcing and dual channel with an unreliable alternative supplier. Prod. Oper. Manag..

[43] Chen T., Wang Y.C., Chiu M.C. (2020). Assessing the robustness of a factory amid the COVID-19 pandemic: A fuzzy collaborative intelligence approach. Healthcare.

[44] Fong S.J., Li G., Dey N., Crespo R.G., Herrera-Viedma E. (2020). Composite Monte Carlo decision making under high uncertainty of novel coronavirus epidemic using hybridized deep learning and fuzzy rule induction. Appl. Soft Comput..

[45] Coulthard P. (2020). Dentistry and coronavirus (COVID-19)-moral decision-making. Br. Dent. J..

[46] Melin P., Monica J.C., Sanchez D., Castillo O. (2020). Multiple ensemble neural network models with fuzzy response aggregation for predicting COVID-19 time series: The case of Mexico. Healthcare.

[47] Toğaçar M., Ergen B., Cömert Z. (2020). COVID-19 detection using deep learning models to exploit Social Mimic Optimization and structured chest X-ray images using fuzzy color and stacking approaches. Comput. Biol. Med..

[48] Fu Y.L., Liang K.C. (2020). Fuzzy logic programming and adaptable design of medical products for the COVID-19 anti-epidemic normalization. Comput. Methods Programs Biomed..

[49] Chen T.C.T., Wu H.C. (2020). Forecasting the unit cost of a DRAM product using a layered partial-consensus fuzzy collaborative forecasting approach. Complex Int. Syst..

[50] Wu G., Yang P., Xie Y., Woodruff H.C., Rao X., Guiot J., Frix A.-N., Louis R., Moutschen M., Li J. (2020). Development of a clinical decision support system for severity risk prediction and triage of COVID-19 patients at hospital admission: An international multicentre study. Eur. Respir. J..

[51] Chiu M.C., Chen T.C.T. (2020). Assessing sustainable effectiveness of the adjustment mechanism of a ubiquitous clinic recommendation system. Health Care Manag. Sci..

[52] Chen T.C.T., Chiu M.C. (2020). Mining the preferences of patients for ubiquitous clinic recommendation. Health Care Manag. Sci..

[53] Govindan K., Mina H., Alavi B. (2020). A decision support system for demand management in healthcare supply chains considering the epidemic outbreaks: A case study of coronavirus disease 2019 (COVID-19). Transp. Res. Part E Logist. Transp. Rev..

[54] Yue P., Gizem Korkmaz A., Zhou H. (2020). Household financial decision making amidst the COVID-19 pandemic. Emerg. Mark. Financ. Trade.

[55] Burlea-Schiopoiu A., Ferhati K. (2020). The managerial implications of the key performance indicators in healthcare sector: A cluster analysis. Healthcare.

[56] Yu S.C., Chen H.R., Liu A.C., Lee H.Y. (2020). Toward COVID-19 information: Infodemic or fear of missing out?. Healthcare.

[57] Lystad R.P., Brown B.T., Swain M.S., Engel R.M. (2020). Impact of the COVID-19 pandemic on manual therapy service utilization within the Australian private healthcare setting. Healthcare.

[58] Hanss M. (2005). Applied Fuzzy Arithmetic.

[59] Promentilla M.A.B., Furuichi T., Ishii K., Tanikawa N. (2008). A fuzzy analytic network process for multi-criteria evaluation of contaminated site remedial countermeasures. J. Environ. Manag..

[60] Saaty T.L. (1986). Axiomatic foundation of the analytic hierarchy process. Manag. Sci..

[61] Wedley W.C. (1993). Consistency prediction for incomplete AHP matrices. Math. Comput. Model..

[62] Lima Junior F.R., Osiro L., Carpinetti L.C.R. (2014). A comparison between Fuzzy AHP and Fuzzy TOPSIS methods to supplier selection. Appl. Soft Comput..

[63] van Broekhoven E., De Baets B. (2006). Fast and accurate center of gravity defuzzification of fuzzy system outputs defined on trapezoidal fuzzy partitions. Fuzzy Sets Syst..

[64] PEMANDU Associates the Global COVID-19 Index (GCI). https://covid19.pemandu.org/#main.

[65] Kim K.K., Park S.H., Ryoo S.Y., Park S.K. (2010). Inter-organizational cooperation in buyer–supplier relationships: Both perspectives. J. Bus. Res..

[66] Bensaou M. (1997). Interorganizational cooperation: The role of information technology an empirical comparison of US and Japanese supplier relations. Inf. Syst. Res..

[67] Virusncov.com COVID-19 Coronavirus–Update. https://virusncov.com/.

[68] Biswas T.K., Das M.C. (2020). Selection of the barriers of supply chain management in Indian manufacturing sectors due to COVID-19 impacts. Oper. Res. Eng. Sci. Theory Appl..

